# Modulation of Paternal Care Behaviors in Response to Stream Conditions by Eastern Hellbenders (*Cryptobranchus alleganiensis alleganiensis*)

**DOI:** 10.1093/iob/obaf007

**Published:** 2025-02-28

**Authors:** R S M O'Brien, J Groffen, A A Dayer, W A Hopkins

**Affiliations:** Department of Fish and Wildlife Conservation, Virginia Tech, Blacksburg, VA 24061, USA; Department of Fish and Wildlife Conservation, Virginia Tech, Blacksburg, VA 24061, USA; Department of Fish and Wildlife Conservation, Virginia Tech, Blacksburg, VA 24061, USA; Department of Fish and Wildlife Conservation, Virginia Tech, Blacksburg, VA 24061, USA

## Abstract

The rapid environmental changes associated with the Anthropocene mean that flexible behavioral responses may be a critical determinant of animals’ resiliency to anthropogenic disturbance, particularly for species with long generation times and low vagility. One type of behavior that exemplifies this potentially important flexibility is parental care. Eggs and juvenile animals are sensitive to environmental stressors, and the ability of parents to adjust care behaviors to buffer their offspring from rapidly changing conditions may be critical to successful reproduction. In this study, we explore the role of parental care in buffering eggs from anthropogenic stressors in the long-lived, fully aquatic eastern hellbender salamander (*Cryptobranchus alleganiensis alleganiensis*). Using custom-designed infrared cameras installed in underwater artificial shelters in a natural stream, we describe hellbender paternal care behaviors in greater detail than has previously been possible, assess the extent to which hellbender fathers buffer their eggs from increasing levels of silt and decreasing concentrations of dissolved oxygen in nesting cavities, and describe the possible trade-offs that hellbender fathers exhibit between paternal care and self-maintenance behaviors. We found that while hellbender parents buffered their offspring from low dissolved oxygen concentrations by increasing parental care, there was an apparent trade-off between parental care and self-maintenance responses to low oxygen. Hellbender fathers did not show evidence of buffering their offspring from the effects of increasing silt or organic material in their nest cavities. We also found that filial cannibalism is a widespread behavior across nests, with almost all fathers exhibiting some cannibalism, although the extent varied widely. Our study indicates that hellbender parents may be able to reduce the impacts of declines in dissolved oxygen concentration on their offspring to a limited extent, but they may be unable to fully protect offspring from increasing silt.

## Introduction

Eggs and juvenile animals are often sensitive to environmental stressors and suffer high levels of mortality as a result (Gross 2005). One means of improving the survival of young animals is parental care, which can help shield (or “buffer”) them from environmental factors until they reach older, more self-sufficient life stages (Dybala et al. 2013; Klug and Bonsall 2014). Parents have been shown to modify their care behavior in response to changing environmental conditions (Potts et al. 1988; Jones and Reynolds 1999; Doody et al. 2006; Kight and Swaddle 2011; Delia et al. 2013; Durant et al. 2019; e.g., Torricelli et al. 1985), but these modifications are not without trade-offs to the parents (Jones and Reynolds 1999). Such trade-offs are in line with evolutionary theory, which suggests that parents must often balance the probability of successful current versus future reproductive events, as well as their own survival and the survival of their offspring (Stearns 2000). Understanding how parents respond to environmental challenges, including balancing trade-offs between self-maintenance and parental care, is increasingly important to informing conservation efforts and understanding how species may respond to the mounting stressors of the Anthropocene.

Freshwater streams represent important, yet understudied systems for studying the effects of anthropogenic change on parental care behaviors. Lotic systems are facing mounting environmental challenges (Reid et al. 2019), and within these systems, species of many taxa including fish (e.g., darters; Knouft et al. 2003; Knouft and Page 2004), insects (e.g., water bugs; Smith 1976), and amphibians (e.g., Japanese giant salamanders; Okada et al. 2015) exhibit parental care that could buffer sensitive juvenile life stages from the effects of environmental variability. However, relatively little research has been done on parental care in relation to environmental conditions in lotic systems.

There are two threats in particular that are likely to be relevant to parental care in freshwater streams. The first of these is declining dissolved oxygen, which is a growing concern due to rising stream temperatures from global climate change (Rajesh and Rehana 2022), stream bank clearing (Davies and Nelson 1994), and increased nutrient loading (Smith 2003). Because many freshwater streams have high dissolved oxygen concentrations compared to other aquatic systems (e.g., Blaszczak et al. 2023), even modest changes in dissolved oxygen may adversely affect species specialized to highly oxygenated environments. Hypoxia can lead to developmental delays and mortality in eggs and young aquatic animals (Jacob et al. 1984; Mills and Barnhart 1999; Pollock et al. 2007), but parental care can improve oxygenation in aquatic systems through behaviors such as fanning, where the parent waves their tail back and forth over the eggs (Jones and Reynolds 1999). Hypoxia and parental responses to it have been well studied in marine and lake systems, but the importance of hypoxia to biota in lotic systems is understudied (Blaszczak et al. 2023). Another growing threat to freshwater streams is siltation, which is being exacerbated by deforestation (Davies and Nelson 1994) and increasingly intense rainfall events due to climate change (Trenberth 2011). Among many other effects, silt can smother aquatic eggs and larvae for a variety of species (Potts et al. 1988; Kemp et al. 2011) and fill in the interstitial spaces upon which small and/or young animals depend (Nickerson et al. 2003). However, nest cleaning, where the parent removes silt from the nest through behaviors such as pushing at the substrate (Potts et al. 1988), may reduce the threat of silt to developing eggs.

**Fig. 1:**
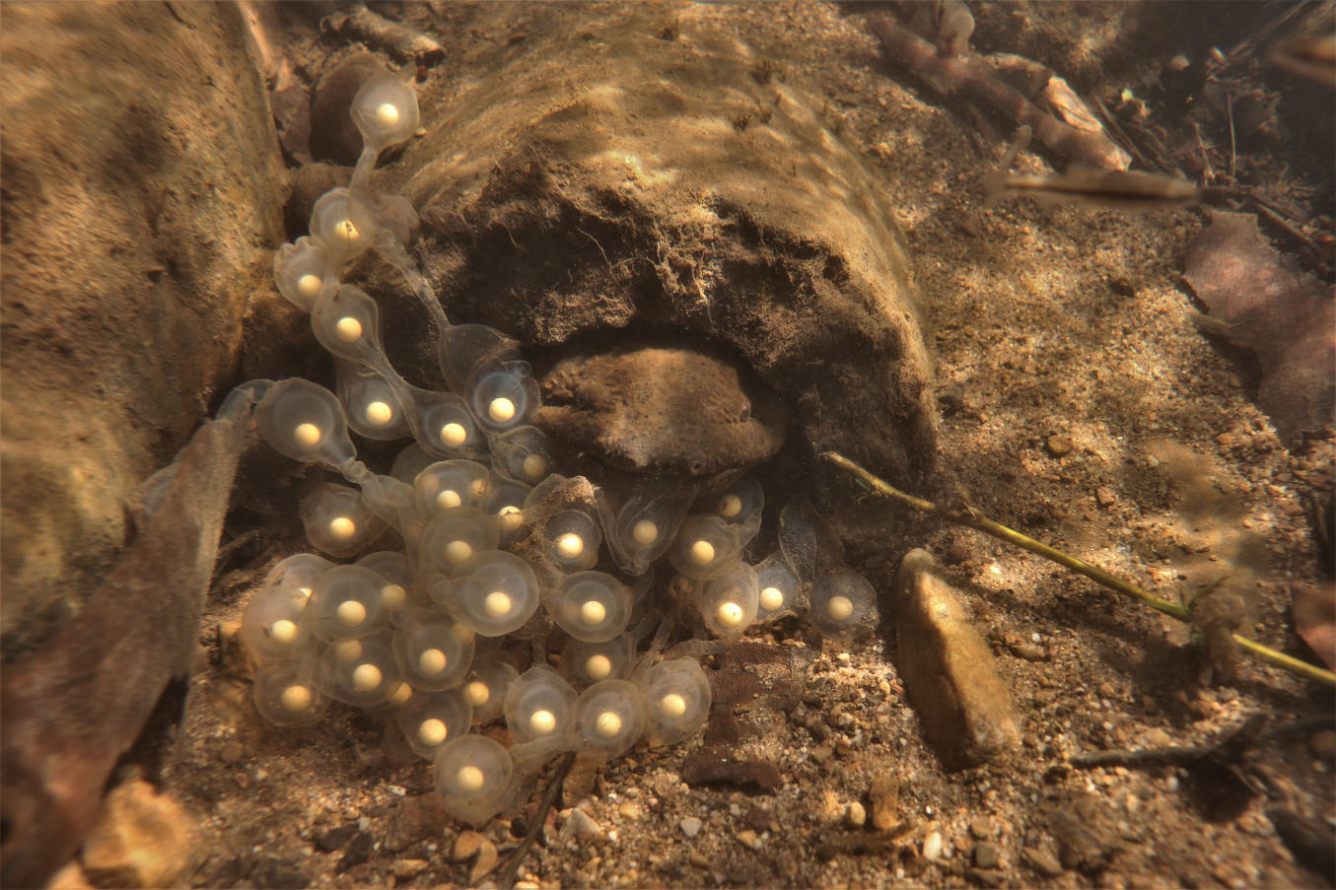
Hellbender clutches consist of long strands of eggs connected by an enveloping jelly. The eggs are typically tied into a knotted form (O'Brien et al. 2024). This figure shows a den master saving an escaping clutch from being swept downstream. Such behavior may be an important parental care behavior. Photo: J. Groffen.

Eastern hellbender salamanders (*Cryptobranchus alleganiensis alleganiensis*, Fig. 1) represent an excellent species for studying the buffering potential of parental care in lotic systems. Eastern hellbenders, and closely related Ozark hellbenders (*Cryptobranchus alleganiensis bishopi*), are long-lived, fully aquatic giant salamanders with a relatively prolonged period of paternal parental care (Nickerson and Mays 1973; Taber et al. 1975; Peterson et al. 1988; Hopkins et al. 2023). Hellbenders’ slow life history strategies and long generation times mean rapid anthropogenic change could outpace their capacity for genetic adaptation (Chevin et al. 2010), which heightens the importance of behavior to this species as a means of responding to novel and/or increasing intensity of stressors.

Hellbenders are highly specialized to inhabit streams and rivers, and typically thrive in cool, well-oxygenated conditions with intact riparian forests (Nickerson and Mays 1973; Jachowski and Hopkins 2018). Adults rely almost entirely on cutaneous respiration (roughly 90% of respiration is aquatic cutaneous respiration; Guimond and Hutchinson 1973), and this, coupled with their large body size, may make them particularly susceptible to threats like deoxygenation. Hellbender eggs are similarly sensitive to changing stream conditions. Hellbenders produce large clutches of hundreds of eggs, and clutches can exceed 1000 eggs when multiple females visit a single nest (Hopkins et al. 2023; O'Brien et al. 2024). This may create high oxygen demand within the nest cavity. Additionally, the eggs are laid in sheltered locations that can collect silt (Nickerson and Mays 1973). As with other aquatic eggs (Potts et al. 1988; Kemp et al. 2011), hellbender eggs are at risk of being smothered by siltation.

Breeding males (hereafter referred to as “den masters”; Takahashi et al. 2017) remain with the eggs from the time they are laid in early fall, through hatching (which occurs roughly 60 days after oviposition), and into the spring when the larvae emerge from the nest (at least 8 months following oviposition; Hopkins et al. 2023). In wild conditions, hellbenders lay their eggs in narrow cavities under large boulders in streams (Nickerson and Mays 1973), but they readily utilize artificial shelters when available (Hopkins et al. 2023). Previous research has shown that during the period of care, male den masters guard the eggs from predators, oxygenate the eggs via tail fanning, and clean the nest (Settle et al. 2018; Unger et al. 2020). Den masters are also known to exhibit partial and whole clutch filial cannibalism during the period of care (Settle et al. 2018; Unger and Williams 2018; Hopkins et al. 2023), but the degree to which partial clutch cannibalism is a parental care behavior (i.e., hygienic cannibalism that removes rotten eggs; Okada et al. 2015) or driven by other factors such as self-maintenance is unknown.

Nest cleaning occurs both during and outside of the reproductive season (O'Brien et al. 2024). During this behavior, males move loose substrate on the floor of the shelter by kicking it with their hind legs, shoveling with the nose, or undulating their hind legs and tail very quickly (Terry et al. 2018; O'Brien et al. 2024). The exact purpose of cleaning is unknown, but it is possible that cleaning behavior serves to remove silt, enlarge and shape the nesting cavity, or to reduce the threat of water mold to eggs by minimizing the amount of organic substrate for mold growth (Terry et al. 2018). The extent to which hellbender fathers modify parental care behaviors such as fanning and cleaning in response to changing stream conditions is unknown.

Hellbender populations have declined throughout their range (Wheeler et al. 2003; Foster et al. 2009; Jachowski and Hopkins 2018; US Fish & Wildlife Service 2018), and reproductive failure during the period of paternal care has been implicated in these declines (Hopkins et al. 2023). For this reason, fully understanding how hellbenders modulate their care in response to environmental conditions is a critical knowledge gap. The goal of our study was two-fold. First, taking advantage of a unique filming approach (described in O'Brien et al. 2024), we provide a general description of hellbender paternal care behaviors to expand fundamental understanding of hellbender natural history. By collecting continuous footage inside the nesting cavity, we were able to provide a more holistic view of behaviors than previous studies, which have been temporally limited and restricted to observations made through the narrow nest cavity opening (Settle et al. 2018; Unger et al. 2020). We then determined whether or not the dissolved oxygen concentrations and silt levels in the nest relate to the den masters’ oxygenating (Okada et al. 2015; Settle et al. 2018) and nest cleaning behaviors (Terry et al. 2018; Unger and Williams 2018). We also document whether dissolved oxygen conditions within the nest cavity trigger a self-maintenance, rocking behavior, which increases the flow of water over the adult's own skin thereby increasing oxygen uptake (Harlan and Wilkinson 1981), and assess whether there are apparent trade-offs between self-maintenance and paternal care behaviors.

## Methods

### Study site

This study took place in a third-order stream in southwestern Virginia, USA, that has an average wetted width of 13 m (range: 8–17 m) and depth of 44 cm (range: 11– 125 cm). The stream is the site of long-term hellbender research (>18 years) and supports a comparatively healthy population of hellbenders (Jachowski and Hopkins 2018). At the stream reach level, our study sites have an average fall (August to November)-specific conductance of 201.0 ± 3.2 μS/cm, total dissolved solids concentration of 0.13 ± 0.002 mg/L, pH of 7.8 ± 0.06, water temperature of 15.7°C ± 0.3°C, and dissolved oxygen concentration of 10.2 ± 0.25 mg/L. The study sites have an average of 67% ± 0.24% upstream riparian forest cover (Yang et al. 2018). This makes the stream comparatively more forested and with better water quality than many streams in Virginia with hellbender populations (Bodinof Jachowski et al. 2016; W. Hopkins unpublished data). However, even within a relatively small stretch of a single stream (measuring 13 river kilometers), the habitat quality is variable, with a range of microhabitats (e.g., locations where silt is deposited vs. scouring zones where it is swept away). This variability enabled us to assess different levels of sediment deposition and dissolved oxygen concentrations across nesting sites with a sufficiently large sample size and without the confounding effects of comparing different streams. Although the variability within the stream was not necessarily indicative of human influence, it enabled us to examine the effects that modest increases in human impact could have on stream systems at a broader scale. For example, human activities are known to increase sedimentation (Leigh and Webb 2006), so by comparing a more sedimented microhabitat to a less sedimented microhabitat, we were able to gain insight into how a stream with higher levels of anthropogenic sediment might influence hellbender parental care.

### Video capture

We filmed den master behavior in underwater shelters consisting of a rectangular chamber with a single tunnel entrance. These shelters were similar to a traditional design (Briggler and Ackerson 2012) that has been in use by our research team since 2012, but they were modified to improve stability in the stream (Button et al. 2020) and to accommodate a microcomputer (Raspberry pi Foundation, Cambridge, UK) attached to a Sainsmart infrared camera (Lenexa, KS, USA) in the shelter lid (O'Brien et al. 2024). We programmed the cameras to record at a frame rate of 15 frames/s and a resolution of 1640 × 1232 pixels. The videos were saved to a data storage device every 15 min, and the computer was programmed to shut down after 48 h to avoid a hard shutdown from battery drainage that could damage the computer. Filming was semicontinuous with short pauses to allow us to exchange batteries, replace data storage devices, and restart computers. Longer pauses occurred when circumstances, such as unmanageably high flows, prevented the scheduled battery and USB exchange. The cameras were designed such that the data storage devices and batteries were external to the shelter, enabling us to make exchanges without disturbing the animals (O'Brien et al. 2024).

We recorded footage in a total of 11 shelters, but one shelter was eliminated almost immediately due to an early clutch loss, leaving us with data from 10 shelters to analyze. Filming occurred between 2020 and 2021. Prior to the 2020 breeding season, we installed 10 modified shelters (hereafter referred to as camera shelters) across a range of microhabitats in a stream in southwestern Virginia, USA. The shelters were installed in preexisting arrays of traditional hellbender shelters (Jachowski and Hopkins 2018). Four of these shelters received clutches in 2020. In 2021, we installed an additional five shelters, and we received another two clutches from unique hellbenders that year. To increase our sample size, in 2020 we also exchanged four traditional shelters that received nests with camera shelters 1 month after oviposition had occurred. Pilot work conducted in 2019 demonstrated that we could exchange a den master's shelter without inducing abandonment (*n* = 3 with no abandonment) and that the den masters resumed parental care in a manner similar to that seen in undisturbed shelters (unpublished video data). To exchange a shelter, we removed the den master and his eggs and held them in cold stream water for approximately 1 h. We placed a camera shelter that had been previously weathered in the stream in the same benthic footprint as the original traditional shelter, and we transferred the substrate (i.e., silt, gravel, and leaf litter) from the original, traditional shelter into the newly placed camera shelter. We then returned the male and his eggs to the camera shelter, and all den masters resumed parental care. We did not utilize behavior data for 12 h following the disruption to ensure that it did not influence our results.

We recorded behavior nearly continuously from 30 days (±2 days) after oviposition through 60 days (±2 days) after oviposition (hereafter the 30-day mark will be referred to as mid-embryonic development and the 60-day mark as hatching to reflect developmental milestones of the eggs in the nest). Although den masters remain in the nest for at least 8 months following oviposition, their importance to larval survival at these later stages of development is unknown. For this reason, we ceased our study at a standardized time following oviposition that typically corresponds to hatching. In the event of a nest failure (i.e., the nest contained no viable eggs or larvae), we ceased our behavioral analysis when the nest was definitively identified as having no eggs or larvae by a snorkeler during routine nest checks.

### Water quality measurements

We measured dissolved oxygen concentrations in each shelter using a miniDOT dissolved oxygen logger (Precision Measurement Engineering, Vista, California) affixed to the ceiling of each shelter's nest chamber. We set the loggers to take a reading every minute, and we calculated the average hourly dissolved oxygen every 4 h (to reflect our behavior sampling frequency described later). Times when the shelter was open, such as during routine nest checks, were eliminated from our dataset.

To assess the level of silt and organic material in each shelter, two researchers scored each shelter on these parameters (silt and organic material independently) using a qualitative scale from 0 to 4, with 0 being minimal silt or organic material and 4 being heavy silt or organic material. We did this at mid-embryonic development and hatching, and the scores were averaged across the two reviewers at each measurement interval.

### Behavioral coding

We used a focal sampling approach and coded the first 15 min of den master behavior every 4 h using ELAN software (Max Planck Institute for Psycholinguistics 2024). This resulted in a cumulative 311 h of analyzed footage. A panel of reviewers who were blind to the den master identification coded the videos. These individuals went through several rounds of training with the lead author before their analysis. Additionally, 10% of all videos were crosschecked between reviewers to ensure consistency, and the lead author reviewed the codes for errors (e.g., improperly interpreted behaviors).

Our ethogram (Table 1) was based on previously identified parental behaviors in hellbenders and in closely related *Andrias* species (Okada et al. 2015; Luo et al. 2018; Settle et al. 2018; Terry et al. 2018) as well as novel behaviors that we observed while coding. To assess the effects of shelter water quality on paternal care, we focused on fanning (supplementary video S1), which is thought to oxygenate the eggs; rocking (supplementary video S2), which is considered to be a self-maintenance behavior of adult hellbenders in conditions of low dissolved oxygen and/or high energetic demands (Harlan and Wilkinson 1981); and cleaning (supplementary video S3), which is thought to agitate and remove sediment from the shelter. Behaviors sometimes occurred simultaneously, and for the purposes of this study, we allowed overlapping codes. An exception to this was when recording the den master's position in the shelter, which was exclusively coded as in the shelter tunnel, in the shelter chamber, or absent from the shelter to enable time budget analysis.

**Table 1.** Our ethogram of den master behaviors was based on previously identified parental care behaviors in hellbenders and closely related species and represents a subset of our more extensive ethogram (O'Brien et al. 2024)

**Behavior**	**Description**
Fanning	A lateral undulation of the tail
Cleaning	Movement of loose substrate using the hind legs in a kicking motion or movement of loose substrate using the nose
Rocking	Lateral and vertical movement of the body while all limbs remain grounded
Striking	Rapid forward lunge while all four feet remain grounded
Charging	Rapid movement toward and through the tunnel
Absent	Male is absent from the camera's field of view after exiting the tunnel and his subsequent return is face first
Occupying chamber	Male is in the chamber without his face in the tunnel
Occupying tunnel	Male's face or entire body is in the tunnel
Agitating	Vigorous movement of the clutch using the head, typically by biting clutch.
Cannibalism	Confirmed consumption of apparently healthy eggs
Hygienic cannibalism	Confirmed consumption of apparently unhealthy eggs
Other behaviors	Vomiting crayfish claws Struggling with unseen object in tunnel Nibbling at eggs Moving eggs with body

### Analyses

To provide a general description of hellbender parental care behavior, we qualitatively summarized our observations of hellbender paternal care and self-maintenance behaviors, and calculated time budgets for the den master's position in the shelter (tunnel, chamber, or absent). We also calculated the percent time that the den masters spent on a few additional behaviors, including rocking and fanning. However, these were not coded exclusively (i.e., codes could overlap), and thus should not be considered a time budget analysis. Nonnesting hellbenders are known to exhibit diel and seasonal differences in the timing of their observable activity outside of boulder cover and bedrock crevices (Humphries 2007; Noeske and Nickerson 2018). However, nothing is known about diel patterns of behavior in nesting hellbenders. Thus, we also used two tailed *t*-tests to compare nocturnal and diurnal levels of noteworthy individual behaviors including standing in the tunnel, rocking, nest cleaning, egg fanning, charging, leaving the shelter, and striking at targets outside the tunnel. For behaviors with short durations (such as charging), we compared the average total number of bouts of behavior that we observed per night or per day in the analyzed footage. For longer duration behaviors (such as fanning), we compared the average total amount of time animals undertook the behavior per night or per day in the analyzed footage. For simplicity, we considered any behavior between 7:00 and 19:00 to be diurnal and anything between 19:00 and 7:00 to be nocturnal.

Using R 4.2.1 (for this and all other statistical analysis, R Core Team 2022), we tested the effects of dissolved oxygen concentration on the percent time that hellbenders spent fanning their eggs or rocking using zero-inflated linear mixed models with β distributions (Brooks et al. 2017). We included the specific shelter (shelter ID) as a random effect in the models, but we found no effect of coders on our results (*P* ≥ 0.20 for all tested behavior codes), so we did not include this as a factor in our models. For the model of percent time spent fanning, we included percent time spent rocking as a covariate, and for the model of percent time rocking, we included the percent time fanning as a covariate. This approach enabled us to determine not only if males modulated their parental care behaviors and self-maintenance behaviors in response to lower dissolved oxygen levels, but also to test for evidence of a trade-off between rocking and fanning.

To determine whether den masters cleaned more in shelters with more silt and/or organic material, we used a zero-inflated negative binomial model that included both silt and organic material as fixed effects and included shelter as a random effect. Because sediment conditions in shelters can change rapidly, we used only the 5 days of behavior prior to or following (depending on the sampling date) the silt or organic material scoring to analyze the effect of shelter sediment on parental care.

### Ethical note

We sought to minimize any risk of clutch abandonment and reduce den master stress by limiting handling time to that strictly necessary to achieving our research goals. We piloted any novel techniques (e.g., shelter exchanges, filming, and use of larger shelters) on a subset of males and ensured that we did not detect any adverse effects prior to undertaking them on a larger number of animals. Any time an animal was removed from the water, we kept them in cool stream water that was refreshed as needed to ensure that it remained at an appropriate temperature and was free of fecal waste. We painted the external surface of all artificial shelters with nontoxic concrete stain to reduce their visibility in the stream and to minimize the risk of disturbance by members of the public. We also soaked the shelters in a cattle tank for up to 8 weeks prior to installation to remove residual toxins from the concrete. Finally, we weathered the shelters in the destination stream for up to 3 months prior to installation to remove any residual chemicals and encourage algal growth and sediment staining that would further reduce visibility in the stream. All work was done under state collecting permits administered by the Virginia Department of Wildlife Resources, and used procedures approved by the Virginia Tech, IACUC (Protocols #19-147 and #22-165).

## Results

In total, we obtained 9109 h of paternal care video footage on 10 nests guarded by 10 unique den masters across two breeding seasons (*n* = 8 and 2 nests for 2020 and 2021, respectively). After subsampling the data, removing uncodable footage, and limiting the footage to the appropriate time frame, we were left with between 9.23 and 38.35 h of coded behavior per shelter, with an average of 29.59 (standard error [SE] = 2.48) h per den master. The variability in coded video footage across nests was due to nest failure and conditions such as rain events making recording impossible.

Four nests that we included in our analysis and one that we eliminated failed during our period of study. These nest failures were unlikely the result of our study design, as a large-scale study of hellbender nesting ecology has found similarly high baseline rates of nest failure (Hopkins et al. 2023). Of the four failed nests that we included in our analysis, one nest failed by 1 month following oviposition and three failed by hatching, providing us with sufficient data to include them in our results. These nests did not have rotten eggs or larvae, but rather had no eggs or larvae present at the time of failure. We were unable to definitively confirm the cause of nest failure in these nests. However, one shelter seemed to have lost its clutch during a high flow rain event, as the eggs were visible before but not after a rain event during which the water became very turbid. The others were likely the result of whole clutch filial cannibalism (as described in Hopkins et al. 2023) because cannibalism was observed on their videos. However, we could not definitively assert this as the cause of complete nest failure. In addition to the 10 nests that we included in this study, we originally had an eleventh nest. However, this nest failed shortly after oviposition when the den master accidentally wrapped a strand of eggs around his leg and pulled it out of the tunnel while exiting the shelter. The stream current subsequently dragged the entire clutch out of the shelter and downstream. We had minimal footage before this occurrence, so this nest was eliminated from our study.

### General observations of behavior

Positionally, males primarily spent their time in the tunnel of the shelter (mean = 76.3%), which put them between their clutch of eggs and the only entrance to the nest cavity. On several occasions, we observed den masters apparently struggling with unseen targets while in the tunnel in potential shelter guarding behavior. Of their remaining time, males spent most of it in the chamber (mean = 19.6% of their time) and they left the shelter only rarely, spending an average of 4.2% of the analyzed time out of the shelter (range = 0–16.5% of analyzed video time). Only three males were absent for more than 2% of the analyzed time, and two of those three nests failed. The average amount of time a male was absent from the shelter was also positively correlated with the average number of bouts of cannibalism (β =1.19, SE = 0.24, T = 4.95, *P* = 0.001), suggesting that these males may have been less invested in parental care.

We definitively identified filial cannibalism of apparently healthy eggs by 5 of the 10 den masters, including males that had both successful (*n* = 3) and unsuccessful (*n* = 2) nests. These instances consisted of the male eating one to a few eggs at a time, which he bit and jerked free from the clutch. For all five of these males, we had sufficient visibility to clearly see the eggs that the den master ate and visually assess that they were developing properly at the time of consumption (dead embryos are visually distinct). We also were able to observe swallowing motions. In one of these shelters, we additionally confirmed filial cannibalism of an opaque, obviously rotten egg, suggesting hygienic cannibalism also occurs (supplementary video S4). In three shelters, we could not definitively identify cannibalism, but we nonetheless observed what was very likely egg consumption. In these cases, we observed body and clutch movements indicative of cannibalism, and in most instances, we saw the male with an egg strand in his mouth. However, due to limits in the camera's field of view or obstructions to the line of sight, such as other eggs, we were not able to clearly see the egg being consumed to classify the cannibalistic event as hygienic or otherwise. In these cases, we also were not always able to confirm that the egg was swallowed. We observed several instances of hellbenders taking eggs into their mouths and spitting them out, so although the animal's behavior may have suggested cannibalism, we refrained from classifying them as cannibals due to this uncertainty.

In addition to males biting the eggs and spitting them back out, we observed other noncannibalistic interactions with the eggs. For example, we recorded den masters nibbling at the eggs and the enveloping jelly connecting eggs without any apparent attempt to eat them (supplementary video S5). Males also frequently crawled under and through the clutch and nuzzled the eggs with their snout. At times, males physically moved the clutch's position in the shelter by pushing it with the side of their torso (supplementary video S6). We also observed males agitating the clutch by biting it and shaking it back and forth. This behavior was not an immediate precursor to cannibalism, but rather an independent behavior. Although its purpose remains unclear (it has been suggested as a behavior to prevent yolk adhesion; Okada et al. 2015), we did observe silt being shaken free from the clutch during some periods of agitation, suggesting that it may, among other possible functions, serve to remove silt from the surface of the eggs.

**Fig. 2:**
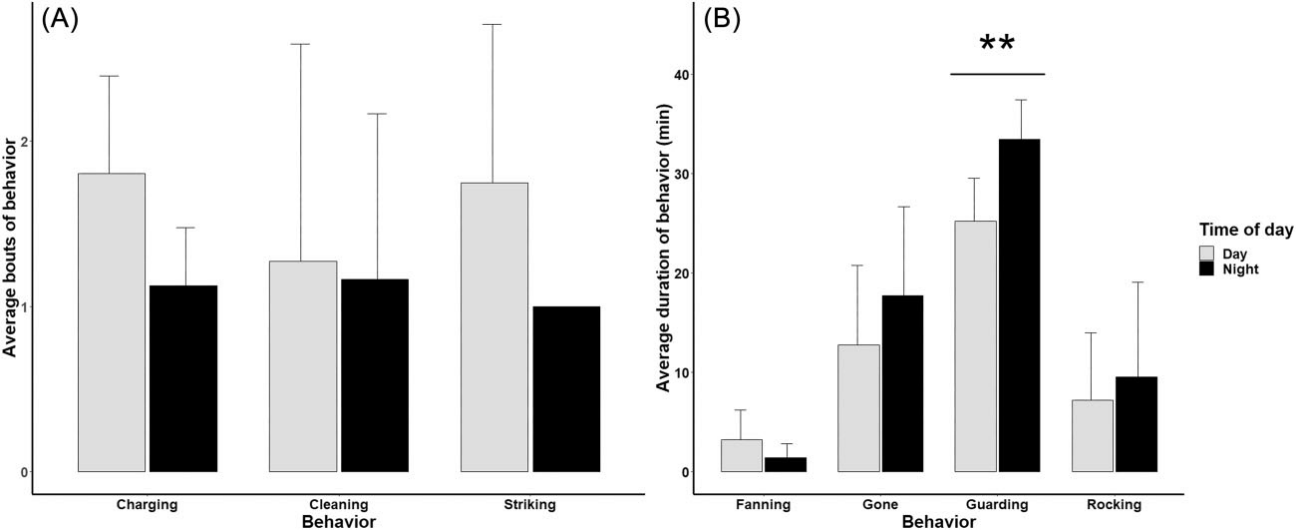
Comparison of nocturnal and diurnal behaviors for male eastern hellbenders during the parental care period. (**A**) The average total number of bouts per animal during the period of recording. (**B**) The average total amount time (min) spent on the behavior per animal during the analyzed period of recording. Error bars show standard deviations. Note that these figures show the average for all analyzed footage per day or night and do not reflect the average amount of time for the entire night/day. **A significant difference between diurnal and nocturnal behavior.

We did not find a clear nocturnal/diurnal bias in behavior in the den masters we recorded, likely due in part to the high variation we saw across animals combined with our small sample size (Fig. 2). The only significant difference in behavior we found was a small difference in the amount of time the den master spent in the tunnel, with males spending significantly more time guarding the tunnel at night than during the day in our analyzed footage ($\bar{\rm x}$_night_ = 33.43, SE_night_ = 1.26 min; $\bar{\rm x}$_day_ = 25.2, SE_day_ = 1.36 min, *P* < 0.001, Fig. 2B).

We observed between zero and four strikes at targets outside the tunnel by individuals during the course of filming and between zero and seven charges (we did not observe striking in five individuals or charging in two individuals). Several of the strike events were accompanied by biting and swallowing, suggesting that they were successful attempts at capturing prey. However, the prey was always consumed in the tunnel and out of the camera's field of view, so we were unable to confirm its identity. In field palpations, we were often able to feel crawdad exoskeletons, and in one instance we recorded an individual vomiting crawdad claws, so this was likely a common target. Charging occurred more frequently than striking and was never immediately followed by striking, suggesting that it was either a defensive behavior and/or a hunting behavior that was often not successful.

### Effects of environmental conditions on paternal care

We observed a range of environmental conditions in the shelters we studied. Our sediment scores varied across shelters with a range of 1.50–3.75 for both silt (mean = 2.6) and organic (mean = 1.92) materials. Although the dissolved oxygen levels within the shelters averaged across the period of study were relatively similar (range of averages = 8.20–9.56 mg/L, mean = 9.06 mg/L), there was meaningful variation across shelters in how consistently the dissolved oxygen remained at a high level. While some shelters remained relatively consistent in their high dissolved oxygen levels, others showed strong fluctuations or saw single events that significantly decreased dissolved oxygen to a level that persisted for prolonged periods. This variation in dissolved oxygen is reflected in the 4-h averages of dissolved oxygen concentrations, which ranged from 6.34 to 11.45 mg/L (66.20–108.28% saturated). The probable cause of the fluctuations in dissolved oxygen varied. In some cases, large influxes of organic material likely drove down the dissolved oxygen levels in the shelter. In other instances, the tunnel entrance became partially or completely buried in sediment such that there was limited exchange of freshwater into the shelter. In some instances, the causes of changes in dissolved oxygen concentrations were unclear. We did not find a significant relationship between a shelter's average dissolved oxygen and either its average organic material score (β = 0.19, SE = 0.13, *P* = 0.19) or its average silt score (β = −0.14, SE = 0.20, *P* = 0.51).

**Fig. 3:**
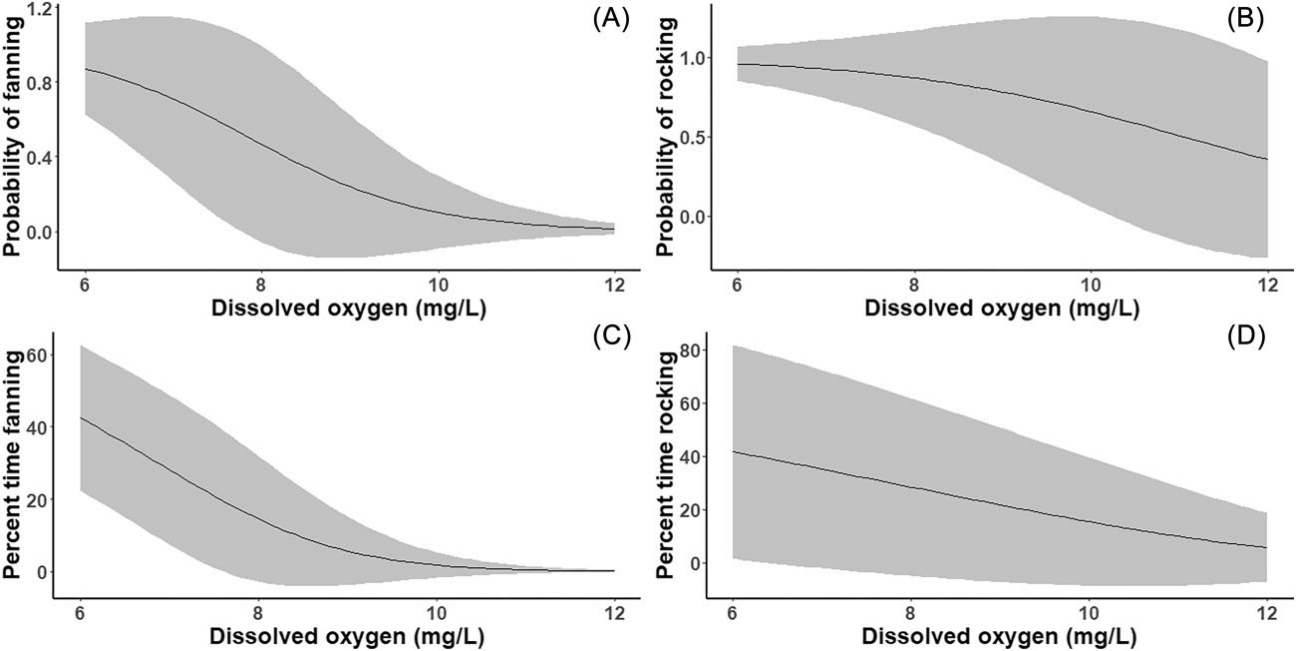
Parental response of eastern hellbender den masters to reduced dissolved oxygen concentrations in terms of both likely parental care behaviors (fanning) and likely self-maintenance behaviors (rocking). We found that average dissolved oxygen concentration had a significant effect on the probability of fanning (**A**) and the percent time spent fanning (**C**) as well as the probability of rocking (**B**) and the percent time spent rocking (**D**). For the purposes of visualization, confidence intervals are displayed using a normal distribution.

Males spent an average of 6.97% (SE = 1.93) of the analyzed time fanning (considered a parental care behavior) and 22.22% (SE = 7.04) of the analyzed time rocking (considered a self-maintenance behavior), and we found that dissolved oxygen influenced both behaviors. Simultaneous rocking and fanning occurred 2.23% (SE = 1.02) of the time. Males were less likely to rock or fan in conditions of higher dissolved oxygen and they spent a smaller percent of their time on these behaviors as dissolved oxygen increased. The model showed a roughly three-fold decrease in the probability of rocking and a roughly 60-fold decrease in the probability of fanning between oxygen concentrations of 6 and 12 mg/L (Fig. 3A and B). The percent time rocking showed a seven-fold decrease and the percent time fanning showed a 313-fold decrease between oxygen concentrations of 6 and 12 mg/L (Fig. 3C and D). We also found some evidence of a moderate trade-off between rocking and fanning, although the effect was nuanced (Table 2). The more time males spent rocking, the less likely they were to fan at all. Likewise, the more time they spent fanning, the less likely they were to do any rocking. However, the amount of time that males spent rocking did not influence the amount of time that they fanned and vice versa.

**Table 2.** Models predicting den master behavior

Dependent variable	Model component	Parameter	Intercept	β	SE	*P*
Percent time spent rocking	Conditional	Dissolved oxygen (mg/L)	1.16	−0.23	0.05	<0.001
		Percent time spent fanning		−0.39	0.22	0.08
	Zero-inflated	Dissolved oxygen (mg/L)	−6.95	0.62	0.10	<0.001
		Percent time spent fanning		0.98	0.41	0.02
Percent time fanning	Conditional	Dissolved oxygen (mg/L)	2.10	−0.37	0.09	<0.001
		Percent time spent rocking		0.43	0.3	0.15
	Zero-inflated	Dissolved oxygen (mg/L)	−8.21	1.02	0.11	<0.001
		Percent time spent rocking		0.95	0.34	0.006
Cleaning bouts	Conditional	Silt	−0.90	−0.16	0.16	0.31
		Organic material		−0.24	0.19	0.20
	Zero-inflated	Silt	−3.02	0.82	0.74	0.27
		Organic material		0.67	0.71	0.34

Zero-inflated models result in two outputs, which we refer to as the zero-inflated component and the conditional component. The zero-inflated component of the model can be thought of as a binomial model where a greater positive correlation indicates a greater chance of a behavior *not* happening, while the conditional model is similar to a traditional regression.

Several rain events occurred during our period of filming, during which the males typically remained in the tunnel of the shelter. These events caused some shelters to accrue sediment and leaves, and for many shelters with high levels of silt and/or organic materials, it was due to these rain events more than a gradual buildup over time. The extent to which this occurred varied across shelters. We did not find any relationship between the levels of silt or the levels of organic material and the amount of cleaning done by the den master (Table 2).

## Discussion

This study provides the most detailed observation to date of hellbender parental care behavior, and it suggests that paternal care may help hellbender offspring withstand some anthropogenic stressors. However, our findings suggest that such behavioral buffering is not without limits, as males only responded to some stressors through increased parental care, and they showed evidence of trade-offs between a likely paternal care behavior (tail fanning) and a likely self-maintenance behavior (rocking) as the intensity of stressors increased.

We found that den masters showed evidence of buffering their offspring from the effects of low dissolved oxygen by increasing their tail fanning behavior, which may be beneficial as anthropogenic activities reduce dissolved oxygen concentrations in lotic systems. Den masters also increased rocking behavior in response to decreasing oxygen concentrations, and we found that the amount of time males spent fanning had a negative effect on whether they did any rocking and vice versa. The egg-aerating effects of rocking and fanning have not been compared, so whether the trade-off we observed represents a trade-off between self-maintenance and parental care is not entirely clear. However, rocking is not thought to be a parental care behavior (Okada et al. 2015). While males are known to rock while alone in the shelter, fanning is seen only during parental care (Settle et al. 2018; O'Brien et al. 2024), suggesting that fanning is a preferable means of oxygenating eggs while rocking is the preferred means of increasing personal oxygen availability. Our findings suggest that there may be a trade-off between parental care and self-maintenance behavior, and that although parents may be able to buffer their offspring from low dissolved oxygen to some extent, this ability is not without limits. Energy expenditure may be an important aspect of this limitation. Although we demonstrated that hellbenders can simultaneously rock and fan, this is a highly exaggerated movement that is likely energetically expensive. The small average percent time that we observed simultaneous rocking and fanning further suggests that it may not be the preferred means of oxygenation. In common gobies (*Pomatoschistus microps*), increased fanning has been shown to increase weight loss in the parent providing care and increase the probability of future nest abandonment (Jones and Reynolds 1999). There may be similar costs in hellbenders that limit their ability to fully behaviorally compensate for declining oxygen concentrations, especially as it relates to simultaneously oxygenating themselves and their offspring.

The trade-off between self-maintenance behaviors and parental care behaviors that we observed may be problematic in the Anthropocene, particularly if degraded water quality triggers males to prioritize self-maintenance over parental care. Prioritizing future reproduction may have grave consequences for individual fitness, population dynamics, and even species survival in the face of chronic deterioration of water quality (Takahashi et al. 2017). However, the environmental factor that might cause hellbender fathers to prioritize future reproductive opportunities over investment in current parental care has received little attention in previous hellbender research (Hopkins et al. 2023) and needs further investigation.

In contrast to their response to changes in dissolved oxygen, we did not find a relationship between the amount of time that males spent cleaning the shelter and the amount of silt or organic material in the nest. Some of our shelters had quite high silt levels, so it is unlikely that the silt was simply insufficient to have a negative effect. Instead, our findings suggest that males do not behaviorally buffer their offspring from the negative effects of increasing siltation or that nest cleaning serves a different purpose in our system. Our findings are in contrast to those of marine sea sticklebacks, which have been found to increase nest inspection and pushing (a type of nest cleaning behavior) in response to increased siltation in experimental studies (Potts et al. 1988).

There are a few possible explanations for why we found no relationship between nest cleaning and silt. One is that nest cleaning does not serve to remove silt from the nests of hellbenders. Previous authors have speculated that nest cleaning could serve to reduce the threat of water mold to eggs by minimizing the amount of organic material that supports water mold or to enlarge and shape the nesting cavity (Terry et al. 2018). However, we found no relationship between organic material and nest cleaning behavior, so this former alternative explanation is not supported by our data. It is also possible that there are other behaviors hellbenders use to remove silt from the eggs, such as the agitation we observed shaking silt free from the clutch, but further investigation is needed to confirm this. Finally, den masters may simply fail to compensate through increasing siltation. Deforestation has the potential to increase the amount of silt in hellbender nesting cavities and if den masters fail to effectively mitigate this challenge through cleaning or other behaviors, greater silt deposition may smother their eggs and larvae.

In addition to assessing parental responses to environmental stressors, we were able to provide a general description of hellbender parental care behaviors through our research, including confirming previous findings and providing new insights into basic paternal care behavior in hellbenders. For example, consistent with previous observations (Settle et al. 2018; Unger et al. 2020), we found that den masters clean the shelter and agitate their clutches and that they primarily spend their time in the tunnel of the shelter and rarely leave. We also made some novel observations. For example, we found that males that leave the nest more frequently are more likely to experience nest failure, and we observed several novel parental care behaviors that occurred within the chamber including crawling over and through the clutch, moving the clutch's position in the shelter, biting at the eggs without obvious intent to pierce them, and taking eggs into their mouths and spitting them out. One possible explanation for these latter two behaviors is that they represent egg grooming, which serves to remove pathogens and reduce infection by mechanically removing fungal spores and/or applying antimicrobial compounds through transferred saliva (Costa 2006). Although not previously described in salamanders, such behaviors have been identified in other taxa such as insects and fish (Smith-Grayton and Keenleyside 1978; Knouft et al. 2003). Given the threat of water mold to hellbender eggs (Smith 1907; Civiello et al. 2019), such behavior may be beneficial to hellbenders. It is also possible that this biting behavior represents an attempt to assess the quality of the eggs as a precursor to hygienic cannibalism (Takahashi et al. 2017).

Previous work on diel patterns of hellbender activity has focused on activity outside of shelters during the nonbreeding season and has identified primarily nocturnal behavior (Noeske and Nickerson 2018). In contrast to this, we found little difference between nocturnal and diurnal behavior patterns during the period of parental care, including in the time that males spent away from the shelter. This difference may be due to differences in diel patterns between the period of parental care and other periods, differences in the age or sex of individuals observed, or our more extensive period of recording, which could have identified behaviors previously difficult to detect. It is also possible that diel patterns are specific to certain streams or drainages. For example, hellbenders have been found to be diurnally active outside of natural shelters in a stream in North Carolina (Humphries 2007), yet more nocturnal in the laboratory and when sourced from other streams (Noeske and Nickerson 2018).

We were unable to definitively identify the cause of nest failure in any shelters except one, whose eggs were lost to the stream current soon after recording began. However, having the clutch swept downstream was likely the cause of failure in another nest whose eggs were present prior to a high flow event but not after. We have observed a den master saving its eggs from being pulled downstream by biting an escaping clutch and pulling it back into the shelter (Fig. 1), suggesting that preventing clutch loss may be a critical parental care behavior. As high flow events continue to increase in intensity as a result of climate change (Tabari 2020), increased impervious surface area, and deforestation (Bradshaw et al. 2007), preventing clutch loss may be critical for helping hellbenders cope with anthropogenic environmental changes.

Our finding that cannibalism occurs in the majority of nests, including successful ones, supports the suggestion that some level of filial cannibalism may be a normal behavior for hellbenders (Hopkins et al. 2023). Although we were able to definitively identify some cannibalized eggs as rotten, all cannibalistic males also appeared to eat at least some apparently healthy eggs, suggesting that the behavior is not entirely hygienic. Based on studies in other species, filial cannibalism may occur in hellbenders for a variety of reasons. One that deserves particular attention is the possibility that filial cannibalism serves to remove eggs that have not been fertilized or have been fertilized by other males (Neff 2003; e.g., Neff and Gross 2001). Den masters are known to experience nest parasitism by smaller males who attempt to fertilize the eggs (O'Brien et al. 2024), so multiple paternity of some clutches is likely. Additionally, hellbenders are facing increasingly high stream salinity as a result of human activities such as agriculture, resource extraction, and land clearing (Kaushal et al. 2018), which is known to negatively influence fertilization success in brook trout (Bonisławska et al. 2015) and may influence hellbenders as well. Recent research has linked full clutch cannibalism to reduced forest cover (Hopkins et al. 2023), consistent with the possibility that degraded stream water chemistry is contributing to nest failure. Because our observations could not definitively identify cannibalism as the cause of nest failure, we were unable to test for a relationship between whole clutch cannibalism and dissolved oxygen or siltation. Future research should attempt to determine the proximate environmental cues triggering hellbender fathers to cannibalize their entire clutch.

By shedding light on the basics of hellbender parental care and uncovering from which stressors den masters may be able to at least partially shield their offspring and from which they may not, our study provides foundational insights into hellbender behavior and reproduction. Additionally, it represents one of the only studies of parental behavioral responses to environmental stressors in freshwater streams. Our results suggest that increasing siltation may be of greater immediate concern than decreasing levels of dissolved oxygen for young hellbenders because adults offer some buffering of the latter through parental care. However, the increased energetic costs of parental buffering behaviors may induce trade-offs, which need further investigation. Our study was conducted in a stream with relatively good water quality and a fairly narrow range of environmental conditions. Future work across a greater range in siltation levels and oxygenation conditions may reveal additional shifts in behaviors and thresholds that induce these shifts. A better understanding of how parents shield their offspring from anthropogenic stressors, as well as better understanding of limitations on their ability to do so, will enable us to better predict how animals respond to rapid environmental change.

## Supplementary Material

obaf007_Supplemental_Files
