# Supplementary material for: Modulation of Paternal Care Behaviors in Response to Stream Conditions by Eastern Hellbenders (Cryptobranchus alleganiensis alleganiensis)
Source: Integr Org Biol. 2025 Feb 28;7(1):obaf007. doi: 10.1093/iob/obaf007 (PMC11925150; doi:10.1093/iob/obaf007)
Supplement: obaf007_Supplemental_Files [file obaf007_supplemental_files.zip › Captions.docx]

Figure 1

Hellbender clutches consist of long strands of eggs connected by an enveloping jelly. The eggs are typically tied into a knotted form (O’Brien et al., 2024). This figure shows a den master saving an escaping clutch from being swept downstream. Such behavior may be an important parental care behavior. Photo: J. Groffen

Figure 2

Comparison of nocturnal and diurnal behaviors for male eastern hellbenders during the parental care period. Panel A depicts the average total number of bouts per animal during the period of recording and Panel B depicts the average total amount time (mins) spent on the behavior per animal during the analyzed period of recording. Error bars show standard deviations. Note that these figures show the average for all analyzed footage per day or night and do not reflect the average amount of time for the entire night/day. ** denotes a significant difference between diurnal and nocturnal behavior.

Figure 3

Parental response of eastern hellbender den masters to reduced dissolved oxygen concentrations in terms of both likely parental care behaviors (fanning) and likely self-maintenance behaviors (rocking). We found that average dissolved oxygen concentration had a significant effect on the probability of fanning (a) and the percent time spent fanning (c) as well as the probability of rocking (b) and the percent time spent rocking (d). For the purposes of visualization, confidence intervals are displayed using a normal distribution.

Table 1

Our ethogram of den master behaviors was based on previously identified parental care behaviors in hellbenders and closely related species and represents a subset of our more extensive ethogram (O’Brien et al. 2024)

Table 2

Models predicting den master behavior. Zero-inflated models result in two outputs which we refer to as the zero-inflated component and the conditional component. The zero-inflated component of the model can be thought of as a binomial model where greater positive correlation indicates a greater chance of a behavior *not* happening, while the conditional model is similar to a traditional regression.
